# Prevalence of Anti-*Toxoplasma gondii* and Anti-*Brucella* Spp. Antibodies in Pregnant Women From Mogadishu, Somalia

**DOI:** 10.3389/frph.2021.672885

**Published:** 2021-08-04

**Authors:** Abdulkarim A. Yusuf, Ahmed A. Hassan-Kadle, Abdalla M. Ibrahim, Mohamed A. Hassan-Kadle, Abdullahi M. Yasin, Maha Khojaly, João L. Garcia, Rafael F. C. Vieira

**Affiliations:** ^1^Vector-Borne Diseases Laboratory, Department of Veterinary Medicine, Universidade Federal Do Paraná, Curitiba, Brazil; ^2^Abrar Research and Training Centre, Abrar University, Mogadishu, Somalia; ^3^College of Medicine and Health Science, Abrar University, Mogadishu, Somalia; ^4^Central Veterinary Research Laboratory, Khartoum, Sudan; ^5^Department of Veterinary Preventive Medicine, Londrina State University, Londrina, Brazil; ^6^Global One Health Initiative, The Ohio State University, Columbus, OH, United States

**Keywords:** foodborne pathogens, toxoplasmosis, brucellosis, abortion, One Health, Somalia

## Abstract

Toxoplasmosis and brucellosis are zoonotic diseases of worldwide distribution. They both cause abortion and infertility in human and animals. Limited data are available about these pathogens in Somali people and their animals. Hence, this study has evaluated the prevalence of anti-*Toxoplasma gondii* and anti-*Brucella* spp. antibodies in pregnant women in Mogadishu, Somalia. Serum samples from 307 pregnant women from Mogadishu, Somalia were tested for anti-*T. gondii* antibodies by Latex Agglutination Test (LAT) and anti-*Brucella* spp. antibodies by Rose Bengal Plate Test (RBPT) and a commercial competitive-ELISA (cELISA). A total of 119/307 (38.76%) pregnant women had a prior history of abortion. A total of 159/307 (51.79%; 95% CI: 46.2–57.35%) pregnant women were seroreactive for *T. gondii* by LAT at different stages of pregnancy. For *Brucella* spp., when RBPT and cELISA were combined 4/307 (1.30%; 95% CI: 0.36–3.30%) pregnant women were seroreactive to anti-*Brucella* spp. antibodies, being 2/307 (0.65%; 95% CI: 0.18–2.34%) by RBPT and 3/307 (0.98%; 95% CI: 0.33–2.83%) by cELISA. Two women were seroreactive for both agents. A high seropositivity to *T. gondii* and low seropositivity to *Brucella* spp. have been found in pregnant women from Mogadishu, Somalia. Considering the high number of abortions in the country associated to the fact that awareness on other zoonotic abortifacient pathogens in Somalis is very low, further studies should be conducted to evaluate the potential causes of abortions.

## Introduction

Toxoplasmosis, caused by *Toxoplasma gondii*, and brucellosis, caused by *Brucella* spp., are important zoonotic diseases with worldwide occurrence (1–3). These zoonotic pathogens may be transmitted from animals to human beings and lead to negative health consequences such as abortion and complete sterility (4).

Brucellosis in humans is commonly caused by *Brucella melitensis* and/or *Brucella abortus*, and is characterized by inflammation of the genitals and fetal membranes, abortions, sterility and lesions in the lymphatic system and joints (4, 5). Endocarditis and neurological outcomes including motor deficits, cranial nerve deficits, sciatica, confusion and/or psychological disturbances, meningitis and seizures are severe clinical presentations of the disease (6). Spontaneous miscarriage and intrauterine fetal death during the first trimesters have also been reported among pregnant women (5, 7). The most common causes of human infection were linked to consumption of unpasteurized dairy products and labor conditions (e.g., veterinarians, slaughterhouse workers, and animal breeders) (4, 8).

*Toxoplasma gondii* is an important food and waterborne opportunistic pathogen that causes severe disease in immunocompromised individuals including pregnant women which may result in abortion, fetal anomaly, stillbirth, fetal growth restriction, and preterm birth (4, 9, 10). Acute phase of the disease during pregnancy also causes congenital toxoplasmosis (11). Several risk factors have been associated with human toxoplasmosis, particularly cat contact and a history of raw or undercooked meat consumption (4). Somali people do not usually keep pets, but stray cats sometimes let into the houses and some households perform their house activities on the ground with a possibility of high risk of contamination (12). Hygienic conditions, socio-economic structure, food and environment can collectively have a notable influence on the diffusion of *T. gondii* (13). Moreover, a previous study has reported the presence of *T. gondii* (3.12%) in raw milk of camel from Iran (14).

Somalia is a tropical developing country in which climatic and living conditions favors the dissemination of zoonotic pathogens. Despite public and economic importance of toxoplasmosis and brucellosis, few data are available in Somali people (12, 13, 15) and their animals (16–20). In addition, there are little or no concerted medical and veterinary efforts to maximize toxoplasmosis and brucellosis detection rates. Therefore, the present study aimed to assess the prevalence of anti-*T. gondii* and anti-*Brucella* spp. antibodies in pregnant women in Mogadishu, Somalia.

## Materials and Methods

### Ethics Statement

This study was approved by the Ethics Committee on Human Research at Abrar University, Somalia (Reference Number AU/ARTC/EC/04/02/2017). The directors of the involved Health Offices gave their permission to conduct the research in their respective facility. All pregnant women that accepted being part of this study provided a written consent to participate.

### Study Design

A cross-sectional study design was conducted from August 2017 and November 2018 to determine the prevalence of anti-*T*. *gondii* and anti-*Brucella* spp. antibodies in pregnant women referred to the Banadir Maternity and Children Hospital or Maternal and Child Health (MCH) clinics in Mogadishu city, Somalia. Facilities were selected based on their specialty in this sector, while the pregnant women were selected on their willingness to cooperate for this study. Participants were informed about the study and a written consent was signed. The age of pregnant women was stratified into groups of 15–30, 31–40, and >40 years old for statistical analysis. All study participants were interviewed using a questionnaire which included demographics and obstetric information comprising age, gestational age and history of abortion.

### Sampling

A total of 307 blood samples including first trimester (gestational age of <14 weeks; *n* = 44), second trimester (gestational age between 14 and 28 weeks; *n* = 53) and third trimester (gestational age >28 weeks; *n* = 210) pregnant women were evaluated. Blood samples (3 mL) were collected by nurses by venipuncture of brachial vein using plain sterile vacutainer tubes and labeled. Samples were kept at room temperature (25°C) until visible clot formation, and then centrifuged at 1,500 × g for 5 min and stored at −20°C until laboratory analysis.

### Serological Diagnosis of Anti-*T. gondii* and Anti-*Brucella* Spp. Antibodies

For the detection of anti-*T. gondii* antibodies, serum samples were screened by a commercial latex agglutination test (LAT) (SPINREACT, S.A/S.A.U Ctra, Santa Coloma, Spain), according to the manufacturers' instructions. The positive reactors were then diluted; two-fold dilution, 1:2 up to 1:128. Sera showing titer of ≥1:2 were considered positive for *T*. *gondii*.

For anti-*Brucella* spp. antibodies detection, serum samples were initially screened by the Rose Bengal Plate Test (RBPT) (CVRL, Khartoum, Sudan) and retested by a commercial competitive-ELISA (cELISA) (Svanova Biotech AB, Uppsala, Sweden), according to the manufacturers' instructions. The optical density (OD) was measured using a wavelength of 450 nm, and samples with a percentage of inhibition (% I) ≥30% were considered positive by cELISA. Samples were considered seropositive for anti-*Brucella* spp. antibodies when the serum tested positive to RBPT and/or cELISA.

### Data Analysis

Data were compiled and analyzed by Statistical Package for Social Sciences (SPSS) version 25 (IBM Corp., Armonk, NY, USA). Either Chi-square or Fisher's exact-test was used to assess association of the age, gestational age and history of abortion with seropositivity of anti-*T*. *gondii* and anti-*Brucella* spp. antibodies. Odds ratio (OR), 95% confidence intervals (95% CI) and *P*-values were calculated, and results were considered significant when *P* < 0.05.

## Results

The majority of pregnant women were found within the age group 15–30 years (85.34%) and two-thirds were presented in the third trimester of gestational age (68.40%). A total of 119/307 (38.76%) pregnant women had a prior history of abortion (Table 1).

**Table 1:** Prevalence of anti-*Toxoplasma gondi*i and anti-*Brucella* spp. antibodies in pregnant women from Mogadishu, Somalia.

**Variable**		**LAT**				**RBPT**		**cELISA**	
		**+/*n***	**Prevalence (%) (95% CI)**	***P*-value**	**OR (95% CI)**	**+/*n***	**Prevalence (%) (95% CI)**	**+/*n***	**Prevalence (%) (95% CI)**
Age	15–30^*^	132/262	50.38 (44.37–56.39)			1/262	0.38 (0.00–2.35)	2/262	0.76 (0.03–2.9)
	31–40	25/42	59.52 (44.47–72.98)	0.272 (χ^2^ = 1.2)	1.4 (0.7–2.8)	1/42	2.38 (0.00–13.44)	1/42	2.38 (0.00–13.44)
	>40	2/3	66.67 (20.25–94.37)	0.575 (χ^2^ = 0.3)	2 (0.2–22)	0/3	0.00	0/3	0.00
Gestational age	1st trimester	24/44	54.55 (40.06–68.3)	0.507 (χ^2^ = 0.4)	1.2 (0.6–2.4)	0/44	0.00	0/44	0.00
	2nd trimester	32/53	60.38 (46.92–72.43)	0.140 (χ^2^ = 2.2)	1.6 (0.9–2.9)	0/53	0.00	0/53	0.00
	3rd trimester^*^	103/210	49.05 (42.36–55.76)			2/210	0.95 (0.03–3.59)	3/210	1.43 (0.29–4.25)
History of abortion	Yes	64/119	53.78 (44.85–62.48)	0.579 (χ^2^ = 0.3)	1.1 (0.7–1.8)	2/119	1.68 (0.08–6.2)	3/119	2.52 (0.52–7.29)
	No^*^	95/188	50.53 (43.45–57.6)			0/188	0.00	0/188	0.00

* *Reference, +, number of positive samples; n, number of samples; 95% CI, 95% confidence interval; OR, odds ratio*.

A total of 161/307 (52.44%; 95% CI: 46.85–57.99%) pregnant women were seroreactive for at least one pathogen. Anti-*T. gondii* antibodies were detected in 159/307 (51.79%; 95% CI: 46.2–57.35%) pregnant women by LAT. The antibody titers to *T. gondii* positive sera were 5 (3.14%), 55 (34.59%), 50 (31.45%), 26 (16.35%), 16 (10.06%), 7 (4.40%), and 0 (0%) by dilution of 1:2, 1:4, 1:8, 1:16, 1:32, 1:64, and 1:128, respectively. Most of pregnant women (34.59%) had antibody titer of 1:4 while higher antibody titers, 1:64, were detected in seven pregnant women serum sample.

Anti-*Brucella* spp. antibodies were detected in two out of 307 (0.65%; 95% CI: 0.18–2.34%) and three out of 307 (0.98%; 95% CI: 0.33–2.83%) pregnant women by RBPT and cELISA, respectively. Only one out of 307 (0.33%; 95% CI: 0.06–1.82%) pregnant woman was seroreactive for *Brucella* spp. by both methods. All pregnant women seroreactive for *Brucella* spp. were in the third trimester of gestational age and had a history of abortion (Table 1).

Two out of 307 (0.65%; 95% CI: 0.18–2.34%) pregnant women were seropositive for both *T. gondii* and *Brucella* spp. The seroprevalence of *Brucella* spp. and *T. gondii* for each variable evaluated is summarized in Table 1.

## Discussion

Toxoplasmosis and brucellosis are zoonotic diseases that may lead to negative health consequences worldwide (1–4). Hence, determining the prevalence of these pathogens among pregnant women is paramount for prophylactic measures toward the susceptible women and reducing the adverse health events toward the seropositive women (21). Since the Somali civil war of the 1990s, no studies have been conducted on the prevalence and importance of toxoplasmosis and brucellosis in human beings from Somalia, a livestock dependent country in East Africa with a population of around 12.3 million (22). Previous studies conducted in the 1980s in the country have showed toxoplasmosis prevalence ranging from 10 to 61.2% in the general human population (12, 13, 23) and 0% in pregnant women (12), while prevalence of brucellosis ranged between 0 and 0.6% (15, 24).

Herein, the overall seroprevalence of brucellosis in pregnant women was low (1.30%), in agreement with the prevalence of brucellosis in animals (1.7%) from Somalia (16). This may be due to the traditional prevention of diseases in livestock farms through culling of animals with proven reproductive problems. A strong association between human and animal seropositivity of brucellosis has been reported in a linked study in Kenya (25). However, the public health associated risk factors of zoonotic pathogens like prevailing tradition of unheated milk consumption and handling of aborted materials and reproductive excretions with bare hands are commonly practiced in Somalia (16), and this should be considered as a potential risk factor for brucellosis and other abortifacients zoonotic pathogens. Moreover, in the present study, all pregnant women seroreactive for *Brucella* spp. were in the third trimester of gestational age and had a prior history of abortion. However, pregnancy complications associated with brucellosis in the country needs further investigation.

The current finding on the prevalence of toxoplasmosis (51.79%) is slightly higher than the previous reports of 43.6% (13) and 40% (12) in Mogadishu, Somalia. However, the present study is similar with previous reports from Kisumu, Kenya (52%) (26), but lower than the findings from Arba Minch, Ethiopia (79.3%) (27). The high seroprevalence of toxoplasmosis found herein may be related to risk factors described previously for the same location (12). Moreover, the differences in the prevalence of toxoplasmosis between studies may also be due to the antigen used (recombinant protein vs. crude antigens).

In the present study, the seroreactivity rates to toxoplasmosis were higher with the age, in agreement with previous studies (4, 27). This may be explained by the longer period of exposure to risk factors (28). Moreover, association between seroreactivity to *T. gondii* and history of abortion was not found (*p* = 0.579). Although the cause of abortion is multi-factorial, previous studies have associated spontaneous abortion in pregnant women and seroreactivity to *T. gondii* (2, 29).

Previous studies have reported the majority of human beings infected by *T. gondii* after birth are asymptomatic, however, some may develop a mild disease or in rare cases, a more severe systemic illness (2). Nonetheless, transplacental transmission of *T. gondii* occurs if women acquire primary infection during pregnancy (30). However, the risk of vertical transmission to the fetus increases from the first trimester (10–24%) to the third trimester (60–90%), but the potential of congenital defect on fetus is more serious with earlier infections (27, 31). In the present study, we found higher rates of seroreactivity to *T. gondii* in the first and second trimesters of gestational age, indicating more serious effects on the fetus if fetal transmission is developed. Unfortunately, no follow-up was conducted to trace congenital transmission of toxoplasmosis and health consequences of seroreactive pregnant women. Screening for *T. gondii* infection during pregnancy is not regularly performed at all maternity hospitals and clinics in Somalia, and most facilities neglect this screening (data not shown). Prompt diagnosis and treatment is essential for the prevention of a possible vertical transmission of *T. gondii* and may minimize the fetal sequelae (21).

There were some limitations in the present study; the serological tests used were for the detection of anti-IgG antibodies to *T. gondii* and *Brucella* spp. Thus, pregnant women seroreactive for *T. gondii* and *Brucella* spp. may have been previously exposed to the agents. Further studies should use diagnostic methods to detect acute infection (IgM serology and/or qPCR) on pregnant women, in order to guide physicians for administering or not the properly therapy. Moreover, Mogadishu city harbors fewer animal populations when compared to other regions of Somalia, and thus, our findings may not represent pregnant women from other regions. However, our findings may still be useful as baseline information for antenatal care in regions where animal rearing is common, and also help to increase awareness of zoonotic diseases affecting maternal and neonatal health.

## Conclusion

A high seropositivity to *T. gondii* and low seropositivity to *Brucella* spp. have been found in pregnant women from Mogadishu, Somalia. Considering the high number of abortions in the country associated to the fact that awareness on other zoonotic abortifacient pathogens in Somalis is very low, further studies should be conducted to evaluate the potential causes of abortions.

## Data Availability Statement

The raw data supporting the conclusions of this article will be made available by the authors, without undue reservation.

## Ethics Statement

The studies involving human participants were reviewed and approved by the Ethics Committee on Human Research at Abrar University, Somalia (Reference Number AU/ARTC/EC/04/02/2017). The patients/participants provided their written informed consent to participate in this study.

## Author Contributions

AAY, AH-K, AI, and MH-K designed the study. AAY, AH-K, MH-K, and AMY collected the data. AAY, AH-K, and MH-K curated the data. AAY, AH-K, AI, MH-K, AMY, MK, JG, and RV carried out the methodology. AAY, AH-K, and RV performed the data analysis. AAY, AH-K, JG, and RV drafted the manuscript. All authors edited and approved the final manuscript.

## Conflict of Interest

The authors declare that the research was conducted in the absence of any commercial or financial relationships that could be construed as a potential conflict of interest.

## Publisher's Note

All claims expressed in this article are solely those of the authors and do not necessarily represent those of their affiliated organizations, or those of the publisher, the editors and the reviewers. Any product that may be evaluated in this article, or claim that may be made by its manufacturer, is not guaranteed or endorsed by the publisher.
